# Prevalence of intestinal parasitic infections and associated risk factors among schoolchildren in the Plateau Central and Centre-Ouest regions of Burkina Faso

**DOI:** 10.1186/s13071-016-1835-4

**Published:** 2016-10-18

**Authors:** Séverine Erismann, Serge Diagbouga, Peter Odermatt, Astrid M. Knoblauch, Jana Gerold, Akina Shrestha, Tarnagda Grissoum, Aminata Kaboré, Christian Schindler, Jürg Utzinger, Guéladio Cissé

**Affiliations:** 1 Swiss Tropical and Public Health Institute, P.O. Box, CH-4002 Basel, Switzerland; 2 University of Basel, P.O. Box, CH-4003 Basel, Switzerland; 3 Institut de Recherches en Sciences de la Santé, P.O. Box 7192, Ouagadougou, 03 Burkina Faso; 4 Kathmandu University, P.O. Box 6250, 45200 Dhulikhel, Nepal

**Keywords:** Burkina Faso, Helminths, Hygiene, Intestinal protozoa, Polyparasitism, Sanitation, Water

## Abstract

**Background:**

Unsafe drinking water, unimproved sanitation and lack of hygiene pose health risks, particularly to children in low- and middle-income countries. This study aimed to assess the prevalence and risk factors of intestinal parasitic infections in school-aged children in two regions of Burkina Faso.

**Methods:**

A cross-sectional survey was carried out in February 2015 with 385 children aged 8–14 years from eight randomly selected schools in the Plateau Central and Centre-Ouest regions of Burkina Faso. Stool samples were subjected to the Kato-Katz and a formalin-ether concentration method for the diagnosis of helminths and intestinal protozoa infections. Urine samples were examined with a urine filtration technique for *Schistosoma haematobium* eggs. Water samples from community sources (*n* = 37), children’s households (*n* = 95) and children’s drinking water cups (*n* = 113) were analysed for contamination with coliform bacteria and faecal streptococci. Data on individual and family-level risk factors were obtained using a questionnaire. Mixed logistic regression models were employed to determine factors associated with intestinal parasitic infections in schoolchildren.

**Results:**

Intestinal parasitic infections were highly prevalent; 84.7 % of the children harboured intestinal protozoa, while helminth infections were diagnosed in 10.7 % of the children. We found significantly lower odds of pathogenic intestinal protozoa infection (*Entamoeba histolytica/E. dispar* and *Giardia intestinalis*) among children from the Plateau Central, compared to the Centre-Ouest region (*P* < 0.001). Children from households with “freely roaming domestic animals” (*P* = 0.008), particularly dogs (*P* = 0.016) showed higher odds of *G. intestinalis*, and children reporting exposure to freshwater sources through domestic chores had higher odds of *S. haematobium* infection compared to children without this water contact activity (*P* = 0.035). Water quality, household drinking water source and storage did not emerge as significant risk factors for intestinal parasitic infections in children.

**Conclusions:**

Intestinal protozoa but not helminths were highly prevalent among schoolchildren in randomly selected schools in two regions of Burkina Faso. Our findings call for specific public health measures tailored to school-aged children and rural communities in this part of Burkina Faso. It will be interesting to assess the effect of water, sanitation and hygiene interventions on the transmission of intestinal parasitic infections.

**Trial registration:**

ISRCTN17968589 (date assigned: 17 July 2015).

**Electronic supplementary material:**

The online version of this article (doi:10.1186/s13071-016-1835-4) contains supplementary material, which is available to authorized users.

## Background

Parasitic infections remain a major public health problem, particularly among children in low- and middle-income countries (LMICs). Several infectious diseases caused by intestinal protozoa (e.g. amoebiasis and giardiasis) or parasitic worms (e.g. schistosomiasis and soil-transmitted helminthiasis) have been classified as neglected tropical diseases (NTDs), as they primarily persist in socially and economically deprived communities [1, 2]. The lack of access to clean water, improved sanitation and adequate hygiene (WASH) are major contributors to the burden of NTDs [3–5]. Among pathogenic agents associated with lack of WASH, water-borne diseases such as amoebiasis or giardiasis cause substantial gastrointestinal morbidity, malnutrition and mortality [6, 7]. It has been estimated that intestinal amoebiasis caused by *Entamoeba histolytica* led to 11,300 deaths worldwide and was ranked fourth in the most fatal parasite-related diseases in 2013 [6, 8]. The prevalence of *Giardia intestinalis* was estimated at 2–3 % in the industrialized world and 20–30 % in LMICs [9]. Water-based diseases (e.g. schistosomiasis) and other parasitic infections constitute another major public health issue in LMICs [10]. Indeed, soil-transmitted helminths were estimated to infect more than one billion people in 2010 with highest prevalence rates observed in school-aged children [11]. It should be noted that most research on parasitic diseases and related morbidity focuses on single species infections. To date, there are no estimates for school-aged children, nor for the entire population, on the global burden of diseases due to polyparasitism of intestinal parasitic infections caused by helminths and intestinal protozoa [1, 11–15].

In Burkina Faso, where polyparasitism is common [16, 17], a deeper understanding of multiple species parasite infections is key for disease control and the reduction of the burden due to these (co-) infections. Whilst health data among under 5-year-old children are collected during national Demographic and Health Surveys (DHS) in Burkina Faso, such as anaemia and *Plasmodium* spp. prevalence, there is a paucity of national health statistics pertaining to school-aged children [18].

In the frame of a project entitled “Vegetables go to School: improving nutrition through agricultural diversification” (VgtS), an intervention study has been conducted in Burkina Faso with the objective of: (i) assessing schoolchildren’s health status at baseline and 1-year follow-up; and (ii) linking a school garden programme to complementary nutrition and WASH interventions, which are described in more detail elsewhere [19]. The present study is part of the VgtS baseline assessment and aims at determining the extent of parasitic infections among children aged 8–14 years and risk factors for infection. Emphasis was placed on household- and school-level water and sanitary conditions, individual hygiene behaviours, and demographic, environmental and socioeconomic characteristics in the Plateau Central and Centre-Ouest regions of Burkina Faso.

## Methods

### Study design and participants

We conducted a cross-sectional survey in February 2015 as part of the VgtS project (cluster randomised trial) in Burkina Faso. The study design is described in detail elsewhere [19]. In brief, eight schools out of the 30 VgtS project schools in Burkina Faso were randomly selected and a random sample of children was invited to participate in the current study.

Our sample size was calculated with regard to the association between the prevalence of intestinal parasitic infection and level of risk in children aged 8–14 years. We assumed a prevalence of intestinal parasitic infections of at least 40 % [20], with a coefficient of variation of 10 % across schools, and a proportion of high-risk children being 25 %. We aimed at a power of 85 % to detect a difference in infection rates with *P* < 0.05 between high- and low-risk children for a true odds ratio (OR) of at least 2 and a total of eight schools. A Monte Carlo simulation (5000 iterations) provided a minimal sample size of 400 children (i.e. 50 children per school). In each of the eight schools, 55–60 children (half boys and half girls) were randomly selected, as we assumed that the final sample size would be reduced by 15 % due to non-response and missing data [19]. The inclusion criteria for the study were: (i) children enrolled in school; (ii) age 8–14 years; (iii) parents or guardians providing written informed consent (fingerprint for illiterate parents/guardians); and (iv) children with oral assent to participate in the study. Children’s caregivers who were willing to participate and who had written informed consent were invited to participate in a household questionnaire survey.

### Study sites

**Fig. 1:**
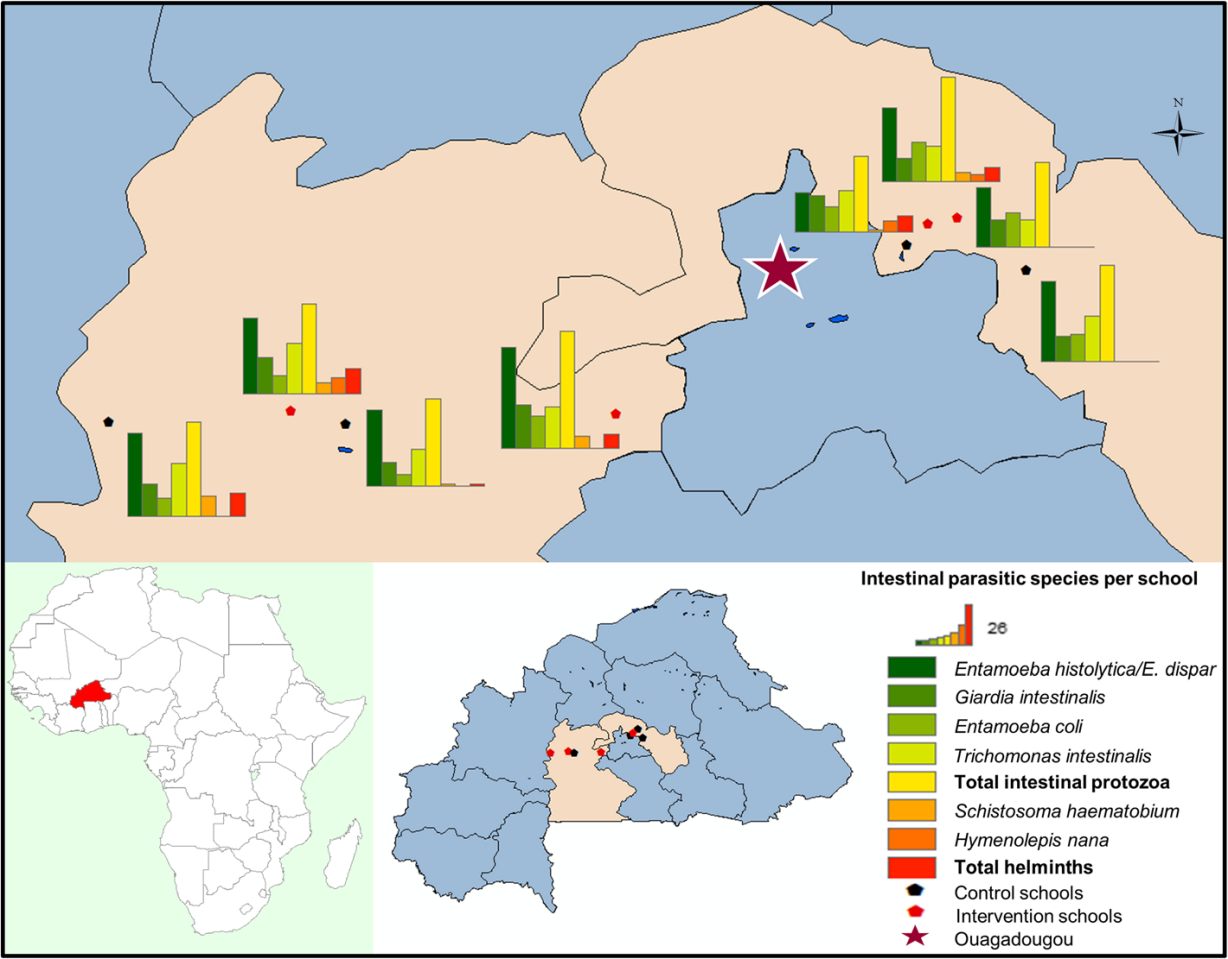
Intestinal parasitic infections among schoolchildren in the two regions of Burkina Faso, February 2015

The study was conducted in the Plateau Central and the Centre-Ouest regions of Burkina Faso. The two regions were selected as VgtS project sites by the local authorities of the Ministry of Education with regards to the objectives of the project and the feasibility of implementing project activities in accessible regions located near the capital Ouagadougou. Both regions lie in the Volta Basin. The Plateau Central region is situated approximately 30–120 km north-east from Ouagadougou and the Centre-Ouest 40–180 km to its south-west (Fig. 1). The climate of the Plateau Central is Sudano-Sahelian, marked by a long dry season lasting from October to May and a short rainy season between June and September. Precipitation is irregular and scant with an annual average of 600 to 800 mm. Drinking water is mainly supplied by surface waters, which are primarily provided by the National Water and Sanitation Authority (Office National de l’Eau et de l’Assainissement, ONEA) of Ziniaré. The hydrographic network of the region is relatively dense but most rivers are temporary. As for the Centre-Ouest, the climate is Sudano-Sahelian with annual precipitation ranging from 700 to 1200 mm. The main water sources used for drinking water are groundwater and water extracted from the Mouhoun River. Communities within our study sites had access to boreholes equipped with manual pumps, as well as improved- and non-improved wells [21].

### Field and laboratory procedures

A questionnaire was administered to children and their caregivers to identify water sources, sanitary and hygiene knowledge, attitudes and practices (KAP), and exposure to unsafe water and sanitation, including potential confounding factors (e.g. household sociodemographic and economic characteristics). Both questionnaires were established according to international guidelines, using standardised questions and amendments made by our research team [18, 22, 23]. The questionnaires were developed in French, translated orally into the local language by research assistants and pre-tested in November 2014 prior to the survey with children and caregivers who did not otherwise participate in the survey (in different schools and villages). Final local adaptations were done before the start of the survey in February 2015. Research assistants entered data directly into tablet computers (Samsung Galaxy note 10.1 N8010) via a data entry mask using Open Data Kit software [24]. Research assistants administered the KAP questionnaire to children at school and visited their caregivers to conduct the household questionnaire at their homes.

Children were asked to provide a fresh morning stool and a mid-morning post-exercise urine sample collected on two consecutive days to assess the presence of soil-transmitted helminths, intestinal protozoa and *Schistosoma* infection. Stool samples were subjected to the Kato-Katz technique (single thick smears, using standard 41.7 mg template), a formalin-ether concentration technique (FECT) for the diagnosis of soil-transmitted helminths (*Ascaris lumbricoides*, hookworm and *Trichuris trichiura*), *S. mansoni*, other helminths and intestinal protozoa (*Blastocystis hominis*, *Chilomastix mesnili*, *Endolimax nana*, *Entamoeba coli*, *E. histolytica* *E. dispar*, *E. hartmanni*, *G. intestinalis* and *Iodamoeba bütschlii*) [25, 26]. Children were considered as positive for a particular infection if at least one of the diagnostic methods revealed a positive result. Urine samples were examined for microhaematuria using reagent strips (Hemastix, Siemens Healthcare Diagnostics GmbH; Eschborn, Germany) and for the presence and number of *S. haematobium* eggs using a urine filtration method [27]. Helminth infection intensity was calculated based on criteria set forth by the World Health Organization (WHO) [28].

Water samples were collected in sterile 250 ml bottles from 30 % of children’s drinking water cups (*n* = 113), 20 % of children’s households (*n* = 95) and from 4–5 community sources per study site (*n* = 37). Samples were transferred to the laboratory in cooled boxes and stored in a fridge at 4 °C before analysis on the same day.

Three bacterial indicators of faecal contamination, namely *Escherichia coli*, faecal coliforms and faecal streptococci, were determined by a membrane filtration technique [29]. Bacterial cells were concentrated on a 0.2 μm Millipore membrane filter, followed by culture on the chromogenic RAPID E. COLI 2 AGAR (BIO RAD) medium to detect *Escherichia coli* and coliform bacteria, or on the bile-esculine-azide medium to identify faecal streptococci. For *Escherichia coli* and coliform bacteria, incubation was performed at 44.5 °C for 24 h. Colonies of *Escherichia coli* appeared violet to pink, while other coliform colonies stained blue. Faecal streptococci appeared as black stains after 24 h of incubation at 37 °C [29].

### Statistical analysis

Kato-Katz thick smear and FECT readings were double-entered into an Excel 2010 spreadsheet (Microsoft; Redmond, USA) and cross-checked. The variable multiple infection was dichotomised in two categories of > 1, and ≤ 1 infections. Prevalences of intestinal parasitic infections, multiple infections and WASH characteristics were compared according to sex, age group (8–11 years and 12–14 years) and region using univariate mixed logistic regression with random intercepts at the level of schools. Mixed logistic regression models were also applied to investigate associations between dependent variables, namely, infections with *H. nana*; *S. haematobium*; pathogenic intestinal protozoa (*E. histolytica/E. dispar* and *G. intestinalis* combined, *E. histolytica/E. dispar* and *G. intestinalis*) and 32 independent variables (e.g. sex and age group). Children with non-pathogenic intestinal protozoa infections (*Trichomonas intestinalis* and *Entamoeba coli*) were excluded from logistic regression analysis. A new variable for hygiene behaviour was created using factor analysis with two conceptually similar categorical variables of: (i) mode of handwashing (handwashing with water and soap, with water only, with ash, and no handwashing); (ii) and its frequency (before eating, after eating, after playing and after defaecation). As the median of the factor score had a relative frequency of over 50 %, the hygiene behaviour of children was categorized as poor, moderate or good depending on whether the score was below, at or above the median. Factor analysis was also used to determine household socioeconomic status (SES). From a list of recorded household assets [30], three factors covering four different socioeconomic domains were retained, including (i) housing wall materials; (ii) roof materials; (iii) floor materials; and (iv) main energy sources used. Each factor score was then categorized into tertile classes. Our multivariate core model included a random intercept at the unit of the school and the categorical exposure variables sex, age group, the three categorical SES-variables from the factor analysis and project region, which were set, *a priori*, as potential confounders. All the other variables were assessed one by one and retained for the maximal model if their *P*-value was < 0.2. The final model was then obtained using backward selection with the same level of 0.2. Associations between infections and risk factors are reported as ORs. Differences and associations were considered statistically significant if *P*-values were below 0.05 and as indicating a trend if *P*-values were between 0.05 and 0.1.

To derive estimates of population attributable fractions (PAF), we ran simple Poisson regression models of the infection outcome variables *Y* on binary exposure variables *X*. Estimates of PAF were then obtained via the formula (*RR* – 1) × *q*/(1 + (*RR* – 1) × *q*) where *RR* denotes the relative risk estimate provided by the Poisson regression model and *q* denotes the prevalence of exposure *X*. Confidence limits of the PAF-estimates were obtained using the same formula. Statistical analyses were done using STATA version 13.0 (Stata Corporation; College Station, USA).

## Results

### Study participation, demographic and socioeconomic profile

Complete datasets were available for 385 children and their caregivers. Of the final study participants, 48.8 % were girls. The age structure of participating children was as follows: 65.2 % were aged 8–11 years and 34.8 % were aged 12–14 years. There was no statistically significant difference in the number of boys and girls in the two age groups (all *P* > 0.05).

Respondents’ demographic and socioeconomic characteristics are summarised in Table 1. Mossi was the predominant ethnic group (68.1 %), followed by Gourunsi with 29.6 %. Most Mossi lived in the Plateau Central, while Gourunsi predominantly lived in the Centre-Ouest region. The houses of children’s families were mainly made of adobe walls (93.3 %), a tin roof (90.4 %) and a clay or mud-type floor (66.2 %). Only 2.3 % of the households were connected to the power grid using electricity or gas; the remaining households used charcoal and firewood as principal energy source. Almost 90 % of children’s caregivers worked in the agricultural sector, while 10.6 % reported non-agricultural sources of income. Domestic animals were kept by 96.4 % of the families, while 63.9 % reported to letting them roam freely within their households. Dogs and goats were particularly common (76.6 and 64.7 %, respectively), followed by cats (39.7 %), swine (28.6 %), cattle (28.3 %), poultry (15.9 %) and sheep (4.4 %). Three-quarters (74.8 %) of the children’s caregivers had no formal education, whereas 15.3 % attended primary school and the remaining 9.9 % reached at least a secondary level of education.

**Table 1:** Characteristics of the study population in the two regions of Burkina Faso in February 2015

Children’s demographic characteristics (*n = *385)		[*n* (%)]	Plateau Central [*n* (%)]	Centre-Ouest [*n* (%)]
Sex				
Girls		188 (48.8)	97 (49.0)	91 (48.7)
Boys		197 (51.2)	101 (51.0)	96 (51.3)
Age of children^a^				
Age group 1 (8–11 years)		251 (65.2)	147 (74.2)	104 (55.6)
Age group 2 (12–14 years)		134 (34.8)	51 (25.8)	83 (44.4)
Ethnicity				
Mossi		262 (68.1)	189 (95.5)	73 (39.0)
Gourunsi		114 (29.6)	1 (0.5)	113 (60.5)
Others (Dioula, Peulh)		9 (2.3)	8 (4.0)	1 (0.5)
Caregiver’s socioeconomic characteristics (*n* = 385)				
Roof material	Simple (natural and baked clay)	37 (9.6)	12 (6.1)	25 (13.4)
	Metal cover	348 (90.4)	186 (93.9)	162 (86.6)
Wall material	Simple (natural clay)	359 (93.3)	182 (91.9)	177 (94.7)
	Baked or cemented clay	26 (6.7)	16 (8.1)	10 (5.3)
Floor material	Simple (clay, sand, mud, straw)	255 (66.2)	115 (58.1)	140 (74.9)
	Baked or cemented clay	130 (33.8)	83 (41.9)	47 (25.1)
Energy used	Simple (charcoal, firewood)	376 (97.7)	191 (96.5)	185 (98.9)
	Electricity and gas	9 (2.3)	7 (3.5)	2 (1.1)
Possession of domestic animals		371 (96.4)	187 (94.4)	184 (98.4)
Animals roaming freely in household		246 (63.9)	124 (62.6)	122 (65.2)
Caregiver’s sociodemographic characteristics (*n* = 385)				
Caregiver’s age^b^				
No formal schooling		288 (74.8)	142 (71.7)	146 (78.1)
Primary education		59 (15.3)	28 (14.1)	31 (16.6)
Secondary or higher education		38 (9.9)	28 (14.1)	10 (5.4)
Main occupation of head of household				
Agriculture		344 (89.4)	180 (90.9)	164 (87.7)
Merchant		8 (2.1)	7 (3.5)	1 (0.5)
Civic service		9 (2.3)	3 (1.5)	6 (3.2)
Others (housework, retirement and no employment)		24 (6.2)	8 (4.0)	16 (8.6)

^a^ = mean age of 11.0 (±0.7) years; 10.8 (±0.1) in the Plateau Central and 11.2 (±0.1) in the Centre-Ouest

^b^ = mean age of 45.0 (±14.2) years; 44.8 (±14.3) in the Plateau Central and 45.2 (±14.1) in the Centre-Ouest

### Prevalence of intestinal parasitic infections

The prevalence of intestinal parasitic infections, stratified by sex, age group and region, are summarised in Table 2. Over 80 % of the schoolchildren were infected with intestinal protozoa. The predominant species was *E. histolytica/E. dispar* (66.5 %), followed by *Entamoeba coli* (37.4 %), *G. intestinalis* (28.1 %), and *Trichomonas intestinalis* (23.4 %). The total prevalence of helminth infections was 10.7 %. *Hymenolepis nana* was the most frequent species (6.5 %), followed by *S. haematobium* (3.9 %) (Fig. 1). Three children were infected with hookworm (0.8 %) and one with *S. mansoni* (0.3 %). Infections with *H. nana*, *S. haematobium,* hookworm and * S. mansoni* were all of light intensity.

**Table 2:** Intestinal parasitic infections among schoolchildren in two regions of Burkina Faso in February 2015

Parasite	Prevalence [*n* (%)]	Sex^a^		Age group^b^		Region^c^	
		F	M	8–11	12–14	PC^d^	CO^d^
Trematodes							
*Schistosoma haematobium*	15 (3.9)	7 (3.7)	8 (4.1)	8 (3.2)	7 (5.2)	8 (4.0)	7 (3.7)
*Schistosoma mansoni*	1 (0.3)	0 (0.0)	1 (0.5)	0 (0.0)	1 (0.8)	0 (0.0)	1 (0.5)
Total *Schistosoma* spp.	16 (4.2)	7 (3.7)	9 (4.6)	8 (3.2)	8 (6.0)	8 (4.0)	8 (4.3)
Nematodes							
Hookworm	3 (0.8)	0 (0.0)	3 (1.5)	2 (0.8)	1 (0.8)	1 (0.5)	2 (1.1)
Cestodes							
*Hymenolepis nana*	25 (6.5)	11 (5.9)	14 (7.1)	13 (5.2)	12 (9.0)	5 (2.5)	20 (10.7)
Total faecal-oral transmitted helminths^e^	27 (7.0)	11 (5.9)	16 (8.1)	15 (6.0)	12 (9.0)	6 (3.0)	21 (11.2)
Intestinal protozoa							
*Entamoeba histolytica/E. dispar*	256 (66.5)	131 (69.7)	125 (63.5)	163 (64.9)	93 (69.4)	110 (55.6)	146 (78.1)
*Entamoeba coli*	144 (37.4)	67 (35.6)	77 (39.1)	93 (37.1)	51 (38.1)	65 (32.8)	79 (42.3)
*Giardia intestinalis*	108 (28.1)	44 (23.4)	64 (32.5)	69 (27.5)	39 (29.1)	49 (24.8)	59 (31.6)
*Trichomonas intestinalis*	90 (23.4)	39 (20.7)	51 (25.9)	51 (20.3)	39 (29.1)	55 (27.8)	35 (18.7)
*Balantidium coli*	1 (0.3)	1 (0.5)	0 (0.0)	0 (0.0)	1 (0.8)	0 (0.0)	1 (0.5)
*Entamoeba histolytica/E. dispar* or *Giardia intestinalis*	290 (75.3)	144 (76.6)	146 (74.1)	182 (72.5)	108 (80.6)	130 (65.7)	160 (85.6)
Total intestinal protozoa^f^	326 (84.7)	161 (85.6)	165 (83.8)	209 (83.3)	117 (87.3)	157 (79.3)	169 (90.4)
Multiple intestinal parasitic infection^g^	206 (53.5)	101 (53.7)	105 (53.3)	124 (49.4)	82 (61.2)	103 (48.0)	111 (59.4)

^a^Significant differences in investigated parasite infection prevalence between boys and girls were found for *Giardia intestinalis* (*P* = 0.05)

^b^ *Trichomonas intestinalis* and multiple parasitic infection prevalence were significantly different between age groups (*P* < 0.05)

^c^Significant regional differences were found for *Hymenolepis nana*, any faecal-oral transmitted helminth, *Entamoeba histolytica/E. dispar, Entamoeba coli, Trichomonas intestinalis, Entamoeba histolytica/E. dispar* or *Giardia intestinalis,* total intestinal protozoa infection, and multiple intestinal parasitic infection (*P* < 0.05)

^d^PC, Plateau Central; CO, Centre-Ouest region of Burkina Faso

^e^The category of total faecal-oral transmitted helminths includes children infected with hookworm and *Hymenolepis nana*. There was one child co-infected with hookworm and *Hymenolepis nana*

^f^Several children were co-infected with intestinal protozoa. The total of this category therefore does not sum up from the separate figures

^g^Multiple intestinal parasitic infection was defined as dichotomous variable, classified as > 1 infection *vs* ≤ 1 infection

**Fig. 2:**
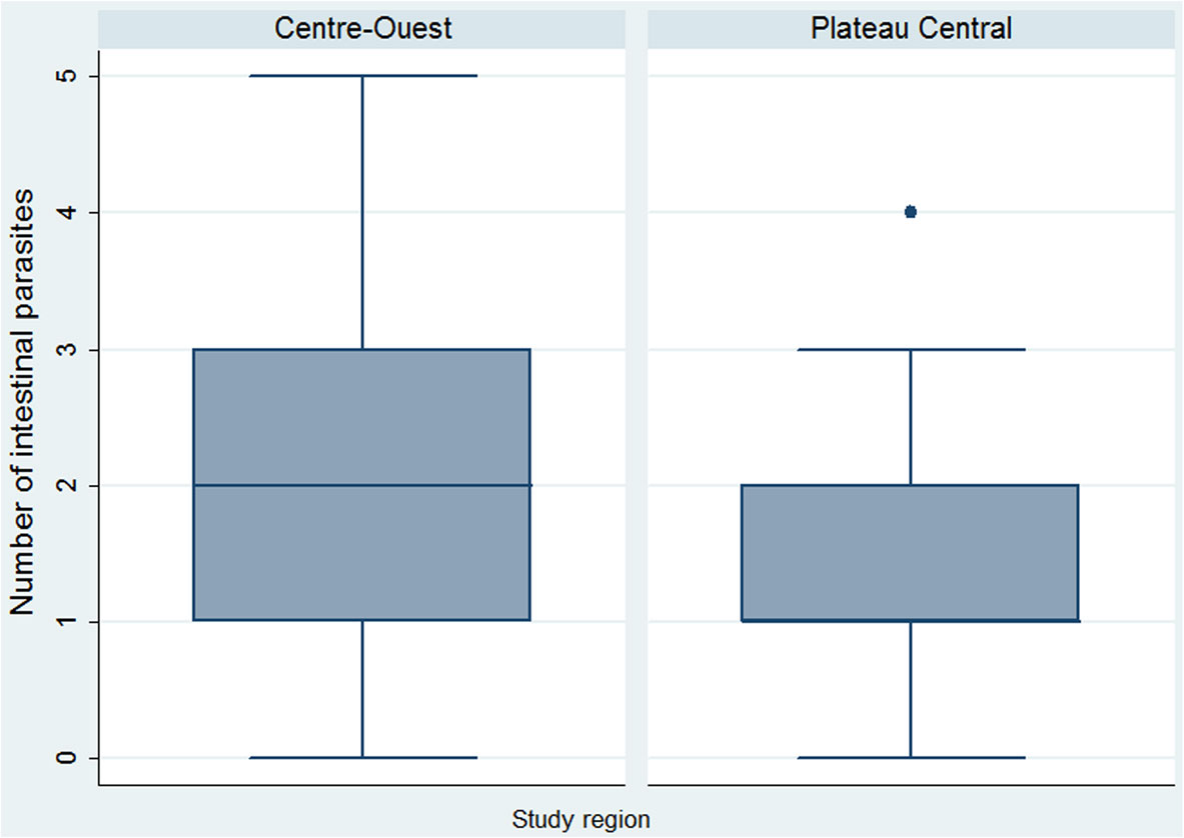
Number of concurrent intestinal parasitic infections, stratified by region among 385 schoolchildren in Burkina Faso. *Box plot*: boxes illustrate the 25^th^ and 75^th^ percentiles (ptile), while the whiskers indicate the adjacent lower and upper values (values which are within 25^th^ ptile – 1.5 * (75^th^ – 25^th^ ptile) and 75^th^ ptile + 1.5 * (75^th^ – 25^th^ ptile), respectively) and values outside these bounds are plotted individually. The median is shown by the line within the boxes

Polyparasitism was common; on average, a study participant harboured 1.7 concurrent parasite species. The maximum number of parasite species found in the same host was five. The large majority of children (86.2 %) were infected with at least one intestinal parasite. Dual (32.5 %), triple (15.6 %), and quadruplicate infections (4.7 %) were also recorded (Fig. 2).

Significant regional differences were observed for the total of intestinal protozoa species found (*χ* ^2^ = 4.68, *df* = 1, *P* = 0.03). There were considerable differences for multiple intestinal parasitic infection profiles among the two regions. Children from the Centre-Ouest were at higher odds of multiple parasitic infections compared to children from the Plateau Central (*χ* ^2^ = 4.98, *df* = 1, *P* = 0.03). The prevalence of infection with *G. intestinalis* was significantly lower in girls compared to boys (*χ* ^2^ = 9.16, *df* = 6, *P* = 0.05; Additional file 1: Table S1). *Trichomonas intestinalis* and multiple parasitic infection prevalence were significantly different between age groups, with children aged 12–14 years at higher odds of infection (*T. intestinalis*: *χ* ^2^ = 3.89, *df* = 1, *P* = 0.05; multiple parasitic infections: *χ* ^2^ = 4.85, *df* = 1, *P* = 0.03).

### WASH behaviours

Based on the findings of the questionnaires conducted with children and their caregivers, most children (87.8 %) reported to wash their hands with soap before eating, while only 22.1 % of children reported doing so after defaecation with an even smaller proportion after playing (7.3 %). Almost 20 % of the children reported defaecating in the fields and bushes. Only 23.1 % of the households had access to an improved latrine, while 21.6 % of their families used a traditional pit latrine and 55.3 % did not own a latrine (Table 3).

**Table 3:** Questionnaire findings and water quality in the two regions of Burkina Faso, February 2015

Children (*n* = 385)	[*n* (%)]	Plateau Central [*n* (%)]	Centre-Ouest [*n* (%)]
Selected KAP indicators^a^			
Handwashing^b^			
Before eating	338 (87.8)	164 (82.8)	174 (93.1)
After eating	55 (14.3)	25 (12.6)	30 (16.0)
After playing	28 (7.3)	12 (6.1)	16 (8.6)
After defaecation	85 (22.1)	41 (20.7)	44 (23.5)
Do not wash hands	16 (4.2)	15 (7.6)	1 (0.5)
Water only	344 (89.4)	183 (92.4)	161 (86.1)
Water and soap	306 (79.5)	153 (77.3)	153 (81.8)
With ash	12 (3.1)	0 (0.0)	12 (6.4)
With mud	1 (0.3)	0 (0.0)	1 (0.5)
Hygiene^c^			
Lower category (1)	56 (14.6)	33 (16.7)	23 (12.3)
Middle score (2)	227 (59.0)	119 (60.1)	108 (57.7)
Best category (3)	102 (26.4)	46 (23.2)	56 (30.0)
Sanitary practices at school^k^			
Using latrines at school	307 (79.7)	181 (91.4)	126 (67.4)
Open defaecation (fields, bush)	71 (18.5)	12 (6.1)	59 (31.5)
Using latrines at home/at teachers’ house	7 (1.8)	5 (2.5)	2 (1.1)
Drinking water^d^			
Drinking water from school	322 (83.6)	174 (87.9)	148 (79.1)
Bringing drinking water from home	239 (62.1)	112 (56.6)	127 (67.9)
Quality of water in children’s drinking cups (*n* = 113)			
Coliform bacteria^k^	101 (89.4)	46 (80.7)	55 (98.2)
*Escherichia coli* ^k^	55 (48.7)	17 (29.8)	38 (67.9)
Faecal streptococci	101 (89.4)	50 (87.7)	51 (91.1)
Safe to drink without prior treatment	3 (2.7)	3 (5.3)	0 (0.0)
Households (*n* = 385)			
Household WASH characteristics^e^			
Type of latrines used			
Flush toilet (i)	0 (0.0)	0 (0.0)	0 (0.0)
VIP latrine^f^ (ii)	14 (3.6)	12 (6.1)	2 (1.1)
Traditional pit latrine (iii)	83 (21.6)	65 (32.8)	18 (9.6)
EcoSan^g^ (iv)	60 (15.6)	33 (16.7)	27 (14.4)
Samplat latrine (v)	15 (3.9)	13 (6.6)	2 (1.1)
No facilities/open defaecation (vi)	213 (55.3)	75 (37.9)	138 (73.8)
Total improved^h^ (i, ii, iv, v)	89 (23.1)	58 (29.3)	31 (16.6)
Total unimproved^i^ (iii, vi)	296 (76.9)	140 (70.7)	156 (83.4)
Preferred source of drinking water during the rainy season			
Private tab	1 (0.3)	1 (0.5)	0 (0.0)
Shared tab	1 (0.3)	1 (0.5)	0 (0.0)
Public tab	25 (6.5)	18 (9.1)	7 (3.7)
Improved source	4 (1.0)	4 (2.1)	0 (0.0)
Un-improved source	8 (2.1)	0 (0.0)	8 (4.3)
Borehole water	249 (64.6)	161 (81.3)	88 (47.1)
Collected rain water	1 (0.3)	1 (0.5)	0 (0.0)
Surface water	3 (0.8)	1 (0.5)	2 (1.1)
Wells	87 (22.6)	14 (7.1)	73 (39.0)
Others	6 (1.5)	1 (0.5)	5 (2.7)
Preferred source of drinking water during the dry season			
Private tab	1 (0.3)	1 (0.5)	0 (0.0)
Shared tab	2 (0.5)	2 (1.0)	0 (0.0)
Public tab	25 (6.5)	18 (9.1)	7 (3.7)
Improved source	4 (1.0)	0 (0.0)	4 (2.1)
Un-improved source	9 (2.4)	0 (0.0)	9 (4.8)
Borehole water	261 (67.8)	168 (84.9)	93 (49.7)
Surface water	0 (0.0)	0 (0.0)	0 (0.0)
Wells	81 (21.0)	8 (4.0)	73 (39.0)
Others	2 (0.5)	1 (0.5)	1 (0.5)
Household drinking water storage			
Open	278 (72.2)	141 (71.2)	137 (73.3)
Pot or canary	290 (75.3)	146 (73.7)	144 (77.0)
Basin or bowl	16 (4.2)	2 (1.0)	14 (7.5)
Canister (plastic jerrican)	59 (15.3)	38 (19.2)	21 (11.2)
Others	18 (4.7)	11 (5.6)	7 (3.7)
No storage	2 (0.5)	1 (0.5)	1 (0.5)
Household drinking water treated prior to consumption^jk^	69 (17.9)	21 (10.6)	48 (25.7)
Water quality of household drinking water (*n* = 95)			
Coliform bacteria	89 (93.7)	42 (89.4)	47 (97.9)
*Escherichia coli* ^k^	61 (64.2)	23 (48.9)	38 (79.2)
Faecal streptococci	88 (92.6)	42 (89.4)	46 (95.8)
Safe to drink without prior treatment	0 (0.0)	0 (0.0)	0 (0.0)
Water quality of community sources (*n* = 37)			
Coliform bacteria	13 (35.1)	4 (22.4)	9 (47.4)
*Escherichia coli*	9 (24.3)	0 (0.0)	9 (47.4)
Faecal streptococci	10 (27.0)	2 (11.1)	8 (42.1)
Safe to drink without prior treatment	22 (59.5)	12 (66.7)	10 (52.6)

^a^Knowledge, attitudes and practices

^b^Multiple responses were possible for the variables characterising the mode (how) and frequency (when) of handwashing

^c^A new variable for hygiene behaviour was created using factor analysis with the mode and frequency of handwashing. Children were classified into three categories with poor, middle and good hygiene behaviours

^d^Multiple responses were possible for the variables characterising the child’s drinking water consumption at school

^e^Water, sanitation, and hygiene

^f^Ventilated improved pit (VIP) latrine is an improved type of pit latrine, which helps remove odours and prevent flies from breeding and escaping. Excreta are collected in a dry pit which has a vent pipe covered with a fly-proof screen at the top

^g^Ecological sanitation (EcoSan) toilets are linked to a closed system that does not need water. The toilet is based on the principle of safely recycling excreta resources to create a valuable resource for agriculture

^h^The improved sanitation category includes all sanitation facilities that hygienically separate human excreta from human contact; i.e. pit latrine with slab, VIP and EcoSan toilets

^i^The unimproved sanitation category includes traditional pit latrines and no facilities (open defaecation)

^j^Households having reported to treat their drinking water through filtration and sedimentation

^k^Significant regional differences were found for children’s sanitary practices (dichotomised variable classified as using latrines *vs*. open defaecation, *χ* ^2^ = 4.67, *df* = 1, *P* = 0.03), water quality of children’s drinking water cups (coliform bacteria, *χ* ^2^ = 5.87, *df* = 1, *P* = 0.02; *Escherichia* coli, *χ* ^2^ = 15.51, *df* = 1, *P* < 0.001); household water treatment (*P* = 0.02); and water quality of household drinking water (*Escherichia coli*, *χ* ^2^ = 8.97, *df* = 7, *P* = 0.003) using mixed logistic regression models with random intercepts at the level of schools

The overall hygiene behaviour, including the modality of handwashing as well as its frequency, and the availability of household latrines was not significantly different across study regions. Yet, statistically significant regional differences were found with regards to children’s sanitary practices; children from the Centre-Ouest practised open defaecation (dichotomised variable with the use of any latrines and open defaecation) more frequently than their counterparts from the Plateau Central (31.5 *vs* 6.1 %; *χ* ^2^ = 4.67, *df* = 1, *P* = 0.03).

Children reported both to drink water at school (83.6 %) and to bring water for consumption from home (62.1 %) (multiple responses were possible). Over 60 % of children’s families were said to use borehole water as drinking water source in the rainy and the dry seasons, as compared to wells, surfaces or collected rain waters. Most households reported storing their water in an open receptacle (72.2 %). Only 17.9 % said they treated their drinking water before consumption. Statistically significant regional differences were found for reported drinking water treatment; households from the Centre-Ouest treated their water more frequently compared to households from the Plateau Central (25.7 *vs* 10.6 %; *χ* ^2^ = 5.53, *df* = 1, *P* = 0.02). The modality of drinking water storage (open *vs* closed) and children’s water exposure through playing, fishing, domestic chores or making laundry did not significantly differ across regions (all *P* > 0.05).

Associations between children’s parasitic infection status and handwashing, sanitary, and hygiene behaviours are summarised in Additional file 2: Table S2. Overall, children with both poor and better hygiene behaviours (first and third category) showed lower odds for any intestinal pathogenic protozoa infections than the middle category, however without these differences reaching statistical significance. Children from households with improved latrines and with soap for handwashing available did not show lower odds for any intestinal parasitic protozoa infection. However, children from households with soap for handwashing available showed lower odds for *H. nana* infection (*P* = 0.23) and *S.haematobium* infection (*P* = 0.06). Children reporting to play, fish and to do domestic chores in water, rivers or watersheds showed higher odds for *S. haematobium* infection*,* but only exposure through domestic chores was statistically significant (*χ* ^2^ = 22.65, *df* = 7, *P* = 0.04). Schoolchildren that reported to drink water from the school source showed significantly lower odds for *S. haematobium* and *H. nana* infection, yet, only the latter was significant in multivariate analysis (*χ* ^2^ = 5.36, *df* = 7, *P* = 0.02). No statistically significant association was found between reported drinking water sources and storages and children’s intestinal protozoa infection status (all *P* > 0.05).

Among domestic animals held by children’s caregivers (cats, cattle, dogs, goats, poultry, sheep and swine), we found a significant association between *G. intestinalis* infection in children and the possession of dogs (*χ* ^2^ = 14.42, *df* = 7, *P* = 0.016; Additional file 1: Table S1). Domestic animals freely roaming in households contributed to 25.6 % of *G. intestinalis* infection in children (95 % CI 4.0–64.4 %), while dogs contributed to 20.0 % of *G. intestinalis* infection in children (95 % CI 2.6–38.8 %). The estimated fraction of *S. haematobium* infection attributable to “any water contact” defined as exposure to freshwater during playing, fishing or doing domestic chores) was 72.0 % infection (95 % CI -45.6–96.1 %).

### Drinking water quality

Table 3 shows the findings from the drinking water quality analysis. About 90 % of water samples from children’s drinking water cups and children’s households were contaminated with both faecal coliform bacteria (89.4 and 93.7 %, respectively) and faecal streptococci (89.4 and 92.6 %, respectively). The proportion of samples contaminated with *Escherichia coli* was smaller; 64.2 % of household drinking water and 48.7 % of children’s drinking water cups were contaminated. Water samples from community sources were less contaminated with faecal coliform bacteria (35.1 %), faecal streptococci (27.0 %) and *Escherichia coli* (24.3 %).

Significant regional differences were found between water samples contaminated with faecal coliform bacteria from children’s drinking water cups (80.7 % in the Plateau Central *vs* 98.2 % in the Centre-Ouest; *χ* ^2^ = 5.87, *df* = 1, *P* = 0.02), and water samples contaminated with *Escherichia coli* from both children’s drinking water cups (29.8 % in the Plateau Central *vs* 67.9 % in the Centre-Ouest; *χ* ^2^ = 15.51, *df* = 1, *P* < 0.001) and households (48.9 % in the Plateau Central *vs* 79.2 % in the Centre-Ouest, *χ* ^2^ = 8.97, *df* = 7, *P* = 0.003).

In univariate logistic regression analysis, household drinking water contaminated with faecal streptococci was associated with a higher odds of total intestinal pathogenic protozoa infections in children (*P* = 0.06), while this association almost collapsed in multivariate analysis (*P* = 0.46). No significant association was found between water quality of community sources and children’s drinking water cups and their status of infection with total pathogenic intestinal protozoa (*P* = 0.79 and *P* = 0.67, respectively).

## Discussion

The findings of the present cross-sectional survey conducted in eight schools in the Plateau Central and the Centre-Ouest regions of Burkina Faso in February 2015 showed that 86.2 % of the participating children aged 8–14 years harboured at least one species of intestinal parasite. Intestinal protozoa were most commonly found; the two predominant pathogenic intestinal protozoan species in the two study regions under investigation were *E. histolytica/E. dispar* (66.5 %) and *G. intestinalis* (28.1 %). Interestingly, we found a significant association between domestic animals roaming freely within households compared to households where domestic animals were kept outside and the prevalence of *G. intestinalis* among schoolchildren. There was a significant association between *G. intestinalis* infection in children and the presence of dogs at the unit of the household. A number of studies have demonstrated *G. intestinalis* as prevalent in both humans and dogs worldwide and have postulated the occurrence of anthroponotic, zoonotic and animal-specific cycles of transmission [31, 32]. The risk of dogs as potential reservoirs would need molecular confirmation [31]. Nevertheless, this finding illustrates the importance of the household environment and highlights the potential role of freely roaming animals, particularly dogs, in the transmission of *G. intestinalis* (PAF of 20.0 %) [31, 33].

*Hymenolepis nana* was the predominant helminth species, however, the overall prevalence was relatively low (6.5 %). Of note, *H. nana* was also the main helminth species found in previous studies in Burkina Faso [16, 34], and is most often found in countries in which sanitation and hygiene are inadequate [35, 36]. We did, however, not find a significantly lower prevalence of *H. nana* in schoolchildren with better hygiene behaviours as would have been anticipated. There was a tendency for lower odds of *H. nana* infections for schoolchildren from families reporting to drink water from borehole sources; yet, these associations lacked statistical significance in multivariate analysis. However, schoolchildren that reported to drink water from the school source showed significantly lower odds for *H. nana* infection in multivariate analysis. It is conceivable that unsafe drinking water contaminated with soil or faeces could act as a carrier of infectious *H. nana* eggs. Yet, the normal mode of transmission is ingestion of the eggs in food contaminated with faeces rather than ingestion of contaminated drinking water [33]. In our study, we did not analyse drinking water for the presence of helminth eggs. Therefore, the association between drinking water source and *H. nana* infection has limited biological plausibility, and cannot be inferred.

The findings from univariate and multivariate mixed logistic regression analyses demonstrated a considerably higher risk of *S. haematobium* infection among children reporting exposure to freshwater sources through domestic chores. This result is in accordance with previous studies, showing a higher prevalence *S. haematobium* infection in children observed to play, work or swim in open water bodies that may contain infected snails [37, 38]. Moreover, children from the Plateau Central showed higher odds of *S. haematobium* infection. Even though this association lacked statistical significance (*P* = 0.64), the observation could be explained by the fact that the Plateau Central holds one of the largest water infrastructures in the country: the Ziga dam (capacity of 200 million m^3^, watershed of Loumbila provided from Nabaouli and Massili Rivers affluent of the Nakambé River, White Volta) and the smaller Loumbila reservoir (36 million m^3^ storage) on the Massili River. The Ziga dam primarily supplies drinking water to the city of Ouagadougou (70 % of its needs in 2008) [21, 39]. Effective solutions to control infection with schistosomes include education and behaviour change and access to abundant supplies of clean water [40]. Yet, water resources are scarce in Burkina Faso, with an average annual precipitation of 600 to 800 mm in the Plateau Central, where the main water sources used for providing drinking water are derived from the Ziga and Loumbila dam. These dams which are closely located around the project schools, may provide suitable snail habitat and may lead to increased risks for school-aged children, particularly through increased water exposure due to their accessibility [41].

We found a significantly lower prevalence of intestinal pathogenic protozoa (in multivariate) and *H. nana* (in univariate) infections in the Plateau Central compared to the Centre-Ouest regions. However, the urbanization rate in the Plateau Central is 7.9 % as compared to 13.2 % in the Centre-Ouest, both of which are lower than the national average (22.7 %). The Centre-Ouest region, with Koudougou as the third largest city in Burkina Faso, plays an economically important role in trade, agriculture and some mining activities [21]. It is therefore interesting to note that the current study found a higher odds of intestinal parasitic infections for children from the economically more developed Centre-Ouest region, as compared to their counterparts living in peri-urban settings in the Plateau Central. Yet, several other factors may explain this observation. First, in the absence of latrines and consistent availability of sanitary infrastructures at schools and households, children from the Centre-Ouest practised open defaecation more frequently than children from the Plateau Central (*P* = 0.02); this can directly lead to faecal contamination (absence of water and cleansing tissues/paper), and thus exposure to intestinal parasitic infections. This has also been described in a previous study conducted among Kenyan schoolchildren, where the presence of tissue/paper or water for anal cleansing emerged as the most important predictor of any soil-transmitted helminth infection [42]. Secondly, water quality also significantly differed between the two study regions; water samples from children’s drinking water cups and households showed significantly higher contamination with *Escherichia coli* in the Centre-Ouest, as compared to the Plateau Central (all *P* < 0.05). Despite the lack of association of faecal contamination of drinking water to children’s parasitic infection status in univariate and multivariate analysis, the presence of faecal coliforms, *Escherichia coli* in water indicates recent faecal contamination and the possible presence of disease-causing pathogens, such as bacteria, viruses and parasites [3, 15, 33]. Lastly, there was a significant difference in reported household water treatment across study regions (higher in the Centre-Ouest compared to the Plateau Central). However, the treatments caregivers reported to use were sedimentation and filtration (with fabric tissue), which may reduce the contents of harmful bacteria but are unlikely to completely remove pathogenic contaminants [33].

While our univariate and multivariate test of associations between schoolchildren’s parasitic infection status and household drinking water source, sanitation and water storage lacked statistical significance, the regional differences found in terms of children’s sanitary practices and safe drinking water are key for explaining the higher prevalence of children’s infection status in the Centre-Ouest. These are most crucial for addressing intestinal parasitic infections in children, in particular for preventing faecal-oral disease transmission [15, 42–44].

The findings of the present study showed that over half of the infected children had polyparasitism and that, on average, a study participant harboured 1.7 intestinal parasite species concurrently. Similar findings were reported among schoolchildren in Côte d’Ivoire and in Kenya, where children were typically infected with an average of two or more species concurrently [45, 46]. We conclude that multiple-species intestinal parasite infections are common in schoolchildren in the Plateau Central and Centre-Ouest of Burkina Faso, partly explained by social-ecological contexts that govern the presence and transmission of intestinal parasitic infections (i.e. climate, proximity to freshwater sources, sanitation and hygiene behaviours) [1, 47].

Lastly, the high prevalence of pathogenic intestinal protozoa infections (75.3 %) compared to that of helminth infections (10.7 %) in this study is in agreement with previous findings in Burkina Faso [17, 34, 48]. Possible reasons for the lower prevalence of faecal-oral transmitted helminths and *Schistosoma* infections among schoolchildren who all had low infection intensity include regular deworming, which reduces both the morbidity caused by these infections and the occurrence of severe complications [49]. The most recent deworming campaign before our survey in 2014 and the implementation of national deworming campaigns since 2004 must be taken into consideration when interpreting our data. They could explain the low intensity of helminth infections found. However, our findings indicate that despite continuous efforts through regular deworming, transmission in the target area is not interrupted [50–52].

The results presented here are of relevance for the control of intestinal parasitic infection in Burkina Faso, justified on the following grounds. First, school-aged children in this part of Burkina Faso are at considerable risk of infection with helminths and particularly intestinal protozoa, including *E. histolytica/E. dispar* and *G. intestinalis*. Hence, measures to prevent children from infection with pathogenic intestinal protozoa, such as hygiene education, improved access to clean water and sanitation at school, should be promoted, as school-aged children represent the main reservoirs for *E. histolytica* and partly *G. intestinalis* transmission [53]. A challenge for controlling intestinal protozoa is the current lack of rapid diagnostic tests to identify pathogenic species and/or pathogenic strains. Harmless commensal intestinal protozoa species are ubiquitous and often morphologically indistinguishable to pathogens; an accurate diagnosis is therefore central to guide treatment and control of intestinal protozoa infections [54]. Second, the burden of disease due to intestinal protozoa infections can be reduced substantially through the improvement of sanitary conditions, adequate excreta disposal, health education and improved hygiene practices [15]. It is, however, unlikely that *E. histolytica/E. dispar* and *G. intestinalis* are eliminated from the environment (cysts are able to survive outside the host for long periods). Third, for this reason, we recommend an integrated control approach to promote water treatment and safe storage. The diversity and integration of different WASH interventions is critical to reduce parasitic intensity, to manage potential risks from pathogenic intestinal protozoa and helminth infections and thus to reduce morbidity in school-aged children [15, 53, 55]. Fourth, we believe that schools are an ideal entry and outreach point for children and their caregivers to provide deworming treatments and individual treatments for children infected with helminths and intestinal protozoa, respectively. Most importantly, for a long-term success, we believe that treatment strategies targeting intestinal protozoa infections need to be integrated with the current national deworming programme and complemented with a diversity of WASH interventions to gain and sustain benefits by reducing reinfection and transmission of intestinal parasitic infections. Finally, cross-sectoral interventions hold promise to make a lasting impact on intestinal parasitic infections by combining school- and community-based initiatives that go beyond WASH and include education and nutrition interventions. An inter-sectoral approach to prevent and control parasitic infections may also benefit schoolchildren’s physical development and educational achievement [56]. The VgtS project provides an opportunity to link the school garden programme to WASH interventions primarily at schools but also at children’s households. A follow-up study conducted after a 12-month intervention period will contribute to understanding the possible effects of these interventions on schoolchildren’s health [19]. Lastly, improvements of WASH infrastructure and appropriate health-seeking behaviour are key to achieve sustained control and elimination of NTDs [57, 58]. Our recommendation of improving WASH infrastructure and appropriate health-seeking behaviour as part of the VgtS project in Burkina Faso would also contribute to ways of moving forward with implementing the Sustainable Development Goals (SDGs) agenda, specifically goal number 6 on “ensuring availability and sustainable management of water and sanitation for all” [59].

There are four main study limitations. First, we pursued a cross-sectional survey in February 2015, and hence, our results only reflect one point in time, i.e. the dry season (November to April). We speculate that the prevalence of parasitic infections might be higher in the rainy season (May to September), when children spend more time outside, work in the fields and might eat more frequently unwashed vegetables and fruits from the garden. Seasonal patterns of intestinal parasitic infections may therefore be underestimated [60, 61]. Second, as we only examined a single Kato-Katz thick smear and FECT from two stool samples of two consecutive days from each child, we underestimated the true prevalence of parasitic infections, due to the low sensitivity of the Kato-Katz technique and urine concentration method [62, 63]. Third, children’s self-reported hygiene behaviours may have resulted in over- or under-reporting of proper hygiene practices [64]. Fourth, the findings presented here are representative for the selected schools in two regions, but cannot be generalised for all of Burkina Faso.

## Conclusions

This study provides new insight into schoolchildren’s parasitic infection status and its associations to household- and school-level WASH conditions among the Plateau Central and Centre-Ouest regions of Burkina Faso. Our findings call for increased public health measures for schoolchildren and rural communities in Burkina Faso. As part of the VgtS project, WASH and health education interventions should be implemented to reduce transmission and reinfection among schoolchildren. Our data will serve as a benchmark for subsequent post-intervention surveys and analysis.

## Additional files

Additional file 1: Table S1. Results from univariate and multivariate logistic regression analysis for *Giardia intestinalis* and *Entamoeba histolytica/Entamoeba dispar. (DOCX 47 kb)*

Additional file 2: Table S2. Results from univariate and multivariate logistic regression analysis for parasitic infection. (DOCX 76 kb)

## Abbreviations

aOR: Adjusted odds ratio
CI: Confidence interval
DHS: Demographic and Health Survey
EKNZ: Ethikkommission Nordwest-und Zentralschweiz
FECT: Formalin-ether concentration technique
IRSS: Institute for Health Sciences Research
KAP: Knowledge, attitudes and practices
LMICs: Low- and middle-income countries
NTD: Neglected tropical disease
ONEA: Office National de l’Eau et de l’Assainissement
PAF: Population attributable fraction
SD: Standard deviation
SDG: Sustainable Development Goal
Swiss TPH: Swiss Tropical and Public Health Institute
VgtS: Vegetables go to School: improving nutrition through agricultural diversification
WASH: Water, sanitation and hygiene
WHO: World Health Organization
