# Supplementary material for: Prevalence of intestinal parasitic infections and associated risk factors among schoolchildren in the Plateau Central and Centre-Ouest regions of Burkina Faso
Source: Parasit Vectors. 2016 Oct 18;9:554. doi: 10.1186/s13071-016-1835-4 (PMC5069922; doi:10.1186/s13071-016-1835-4)
Supplement: Additional file 1: Table S1. — Results from univariate and multivariate logistic regression analysis for Giardia intestinalis and Entamoeba histolytica/Entamoeba dispar. (DOCX 47 kb) [file 13071_2016_1835_MOESM1_ESM.docx]

**Additional file 1: Table S1.** Results from univariate and multivariate logistic regression analysis for *Giardia intestinalis* and *Entamoeba histolytica/Entamoeba dispar*

| **Risk factor** | ***Giardia intestinalis (n = 108)*** | | | | | | | ***Entamoeba histolytica/Entamoeba dispar (n = 256)*** | | | | | | |
| --- | --- | --- | --- | --- | --- | --- | --- | --- | --- | --- | --- | --- | --- | --- |
|  | Univariate log. regression* | | | | Multivariate log. regression** | | | Univariate log. regression* | | | | Multivariate log. regression** | | |
|  | N^f^ | OR | 95% CI | *P* | aOR | 95% CI | *P* | N^f^ | OR | 95% CI | *P* | aOR | 95% CI | *P* |
| **Sex** |  |  |  |  |  |  |  |  |  |  |  |  |  |  |
| Male (197) | 64 | 1.00 |  |  |  |  |  | 125 | 1.00 |  |  |  |  |  |
| Female (188) | 44 | 0.63 | 0.40–1.00 | **0.05** | 0.63 | 0.40–1.00 | **0.05** | 131 | 1.38 | 0.88–2.16 | **0.16** | 1.37 | 0.87–2.15 | 0.17 |
| **Age group** |  |  |  |  |  |  |  |  |  |  |  |  |  |  |
| 8–11 yrs (251) | 69 | 1.00 |  |  |  |  |  | 163 | 1.00 |  |  |  |  |  |
| 12–14 yrs (143) | 39 | 1.08 | 0.68–1.72 | 0.74 | * |  |  | 93 | 1.12 | 0.68–1.85 | 0.67 | * |  |  |
| **Region** |  |  |  |  |  |  |  |  |  |  |  |  |  |  |
| Centre-Ouest (187) | 59 | 1.00 |  |  |  |  |  | 146 | 1.00 |  |  |  |  |  |
| Plateau Central (198) | 49 | 0.71 | 0.46–1.11 | **0.14** | 0.68 | 0.42–1.11 | 0.12 | 110 | 0.35 | 0.18–0.69 | **0.001** | 0.35 | 0.18–0.66 | **0.001** |
| **Hygiene^a^** |  |  |  |  |  |  |  |  |  |  |  |  |  |  |
| Middle third (2) (227) | 62 | 1.00 |  |  |  |  |  | 33 | 1.00 |  |  |  |  |  |
| Lower third (1) (56) | 14 | 0.89 | 0.73–2.03 | 0.73 | * |  |  | 154 | 0.63 | 0.32–1.22 | **0.17** | 0.63 | 0.32–1.22 | 0.17 |
| Higher third (3) (102) | 32 | 1.22 | 0.73–2.03 | 0.45 | * |  |  | 69 | 1.01 | 0.60–1.72 | 0.97 | * |  |  |
| **Drinking water for consumption^b^** |  |  |  |  |  |  |  |  |  |  |  |  |  |  |
| From home (239) | 70 | 1.18 | 0.74–1.87 | 0.49 | * |  |  | 158 | 1.10 | 0.67–1.79 | 0.71 | * |  |  |
| From school (322) | 88 | 0.81 | 0.45–1.45 | 0.48 | * |  |  | 217 | 1.01 | 0.49–2.11 | 0.97 | * |  |  |
| **Water risk behaviours** |  |  |  |  |  |  |  |  |  |  |  |  |  |  |
| No water contact (93) | 27 | 1.00 |  |  |  |  |  | 64 | 1.00 |  |  |  |  |  |
| Playing (5) | 1 | 0.61 | 0.07–5.72 | 0.67 | * |  |  | 3 | 0.45 | 0.07–3.07 | 0.42 | * |  |  |
| Fishing (25) | 8 | 1.15 | 0.44–2.98 | 0.77 | * |  |  | 16 | 0.76 | 0.29–2.03 | 0.59 | * |  |  |
| Making laundry (56) | 9 | 0.47 | 0.20–1.09 | **0.08** | 1.00 | 0.88–1.15 | 0.95 | 37 | 0.83 | 0.39–1.77 | 0.63 | * |  |  |
| Domestic chores (206) | 63 | 1.08 | 0.63–1.84 | 0.79 | * |  |  | 136 | 0.78 | 0.44–1.38 | 0.39 | * |  |  |
| Any water contact^b^ (292) | 81 | 0.94 | 0.56–1.57 | 0.81 | * |  |  | 192 | 0.78 | 0.45–1.34 | 0.37 | * |  |  |
| **Sanitary practices children** |  |  |  |  |  |  |  |  |  |  |  |  |  |  |
| Using latrines at school (307) | 86 | 1.00 |  |  |  |  |  | 204 | 1.00 |  |  |  |  |  |
| Using latrines at home/teacher’s (7) | 2 | 1.03 | 0.20–5.40 | 0.97 | * |  |  | 4 | 1.16 | 0.22–6.09 | 0.86 | * |  |  |
| Open defaecation at school^c^ (71) | 20 | 1.00 | 0.57–1.79 | 0.98 | * |  |  | 48 | 0.74 | 0.36–1.53 | 0.42 | * |  |  |
| **Caregiver’s education** |  |  |  |  |  |  |  |  |  |  |  |  |  |  |
| Never went to school (288) | 83 | 1.00 |  |  |  |  |  | 189 | 1.00 |  |  |  |  |  |
| Primary education (59) | 16 | 0.92 | 0.49–1.72 | 0.79 | * |  |  | 44 | 1.61 | 0.82–3.14 | **0.17** | 1.43 | 0.97–2.10 | 0.07 |
| Secondary education (38) | 9 | 0.77 | 0.35–1.69 | 0.51 | * |  |  | 23 | 1.17 | 0.55–2–52 | 0.68 | * |  |  |
| **Caregiver’s occupation** |  |  |  |  |  |  |  |  |  |  |  |  |  |  |
| Agriculture (344) | 97 | 1.00 |  |  |  |  |  | 232 | 1.00 |  |  |  |  |  |
| Civil service (8) | 1 | 0.36 | 0.04–3.00 | 0.35 | * |  |  | 4 | 0.79 | 0.18–3.59 | 0.76 | * |  |  |
| Merchant (9) | 3 | 1.27 | 0.31–5.19 | 0.74 | * |  |  | 6 | 0.94 | 0.21–4.28 | 0.94 | * |  |  |
| Others^d^ (24) | 7 | 1.05 | 0.42–2.61 | 0.92 | * |  |  | 14 | 0.69 | 0.28–1.75 | 0.44 | * |  |  |
| **Animals^b^** |  |  |  |  |  |  |  |  |  |  |  |  |  |  |
| Possession of domestic animals^g^ (371) | 106 | 2.4 | 0.53–10.91 | 0.26 | * |  |  | 248 | 1.06 | 0.33–3.37 | 0.93 | * |  |  |
| Animals held in the house (246) | 79 | 1.79 | 1.10–2.93 | **0.02** | 2.00 | 1.20–3.34 | **0.01** | 165 | 1.02 | 0.64–1.62 | 0.94 | * |  |  |
| **Household sanitary conditions** |  |  |  |  |  |  |  |  |  |  |  |  |  |  |
| Traditional latrine (213) | 25 | 1.00 |  |  |  |  |  | 52 | 1.00 |  |  |  |  |  |
| No latrines/ open defaecation (83) | 64 | 1.58 | 0.88–2.84 | **0.12** | 1.50 | 0.82–2.77 | 0.19 | 144 | 0.84 | 0.47–1.51 | 0.57 | * |  |  |
| Improved latrine (89) | 19 | 1.59 | 0.80–3.17 | **0.19** | 1.92 | 0.93–3.93 | 0.08 | 60 | 0.93 | 0.48–1.83 | 0.84 | * |  |  |
| Soap for handwashing available^b^ (118) | 33 | 0.99 | 0.61–1.61 | 0.98 | * |  |  | 79 | 1.27 | 0.77–2.08 | 0.35 |  |  |  |
| **Household drinking water rainy season** |  |  |  |  |  |  |  |  |  |  |  |  |  |  |
| Tap source (37) | 13 | 1.00 |  |  |  |  |  | 19 | 1.00 |  |  |  |  |  |
| Borehole water (249) | 68 | 0.69 | 0.33–1.44 | 0.33 | * |  |  | 163 | 1.28 | 0.57–2.88 | 0.54 | * |  |  |
| Well (87) | 25 | 0.74 | 0.33–1.69 | 0.48 | * |  |  | 64 | 1.42 | 0.56–3.63 | 0.46 | * |  |  |
| Rain water, surface water (12) | 2 | 0.37 | 0.07–1.94 | 0.24 | * |  |  | 10 | 1.55 | 0.44–14.73 | 0.30 | * |  |  |
| **Dry season** |  |  |  |  |  |  |  |  |  |  |  |  |  |  |
| Tap source (34) | 13 | 1.00 |  |  |  |  |  | 17 | 1.00 |  |  |  |  |  |
| Borehole water (261) | 68 | 0.57 | 0.27–1.20 | **0.14** | 0.83 | 0.55–1.26 | 0.39 | 175 | 1.27 | 0.53–3.03 | 0.59 | * |  |  |
| Well (81) | 24 | 0.68 | 0.29–1.58 | 0.37 | * |  |  | 58 | 1.04 | 0.37–2.94 | 0.94 | * |  |  |
| Surface water (9) | 3 | 0.81 | 0.17–3.80 | 0.79 | * |  |  | 6 | 0.73 | 0.13–4.11 | 0.72 | * |  |  |
| **Household drinking water storage** |  |  |  |  |  |  |  |  |  |  |  |  |  |  |
| Open^b^ (278) | 77 | 0.94 | 0.57–1.54 | 0.80 | * |  |  | 191 | 1.53 | 0.93–2.51 | **0.10** | 1.58 | 0.95–2.60 | 0.08 |
| Pot or canary (290) | 80 | 1.00 |  |  |  |  |  | 191 | 1.00 |  |  |  |  |  |
| Basin or bowl (16) | 5 | 1.19 | 0.40–3.54 | 0.75 | * |  |  | 13 | 1.63 | 0.44–6.12 | 0.47 | * |  |  |
| Canister (plastic jerrican) (59) | 19 | 1.25 | 0.68–2.28 | 0.47 | * |  |  | 42 | 1.21 | 0.63–2.31 | 0.57 | * |  |  |
| **Household drinking water treatment^b^** |  |  |  |  |  |  |  |  |  |  |  |  |  |  |
| Prior to consumption^e^ (69) | 22 | 1.25 | 0.71–2.20 | 0.44 | * |  |  | 47 | 0.97 | 0.53–1.77 | 0.91 | * |  |  |
| **Water contamination households^b^** |  |  |  |  |  |  |  |  |  |  |  |  |  |  |
| Coliform bacteria (89) | 29 | 2.42 | 0.27–21.64 | 0.43 | * |  |  | 58 | 1.45 | 0.24–8.92 | 0.69 | * |  |  |
| *Escherichia coli* (61) | 22 | 1.83 | 0.71–4.74 | 0.21 | * |  |  | 41 | 1.08 | 0.40–2.89 | 0.89 | * |  |  |
| Faecal streptococci (88) | 29 | 2.95 | 0.34–25.65 | 0.33 | * |  |  | 58 | 1.94 | 0.33–11.39 | 0.46 | * |  |  |
| Safe to drink (34) | 0 | na |  |  | * |  |  | 0 | na |  |  | * |  |  |
| **Water contamination children’s drinking cups^b^** |  |  |  |  |  |  |  |  |  |  |  |  |  |  |
| Coliform bacteria (101) | 25 | 0.67 | 0.18–2.56 | 0.56 | * |  |  | 71 | 2.15 | 0.58–8.00 | 0.25 | * |  |  |
| *Escherichia coli* (55) | 12 | 0.68 | 0.28–1.61 | 0.38 | * |  |  | 41 | 1.60 | 0.66–3.88 | 0.30 | * |  |  |
| Faecal streptococci (101) | 29 | na |  |  | * |  |  | 68 | 0.58 | 0.13–2.51 | 0.47 | * |  |  |
| Safe to drink (61) | 0 | na |  |  | * |  |  | 2 | 1.45 | 0.11–18.62 | 0.78 | * |  |  |
| **Water contamination community sources^b^** |  |  |  |  |  |  |  |  |  |  |  |  |  |  |
| Coliform bacteria (13) | 8 | 1.89 | 0.48–7.49 | 0.36 | * |  |  | 12 | 7.20 | 0.80–65.05 | **0.08** | 5.10 | 0.40–64.25 | 0.21 |
| *Escherichia coli* (9) | 6 | 2.31 | 0.48–11.12 | 0.30 | * |  |  | 9 | na |  |  | * |  |  |
| Faecal streptococci (10) | 7 | 2.92 | 0.62–13.76 | **0.18** | 3.85 | 0.47–31.34 | 0.21 | 9 | 4.50 | 0.49–41.25 | **0.18** | 2.31 | 0.14–39.26 | 0.56 |
| Safe to drink (15) | 9 | 0.35 | 0.09–1.36 | **0.13** | 0.30 | 0.06–1.58 | 0.16 | 14 | 0.27 | 0.05–1.51 | **0.14** | 0.28 | 0.03–2.80 | 0.28 |

^a^A new variable for hygiene behaviour was created using factor analysis with the mode and frequency of handwashing. Children were classified into three categories with poor, middle and good hygiene behaviours.

^b^The odds ratio (OR) refers to the comparison “yes” *vs* “no”

^c^Open defaecation includes the category of defaecating in the bush and behind the latrines

^d^Others’ includes homemakers, retirees and unemployed people

^e^Households reported to treat their drinking water through filtration and sedimentation

^f^N = positive cases

^g^Among domestic animals held by children’s caregivers (cats, cattle, dogs, goats, poultry, sheep and swine), we found a significant association between *Giardia intestinalis* infection in children and the possession of dogs (OR = 2.3, 95% CI 1.26–4.22, *χ^2^* = 7.26, *df* = 1, *P* = 0.007; aOR = 2.1, 95 % CI 1.15–4.00, *χ^2^* = 14.42, *df* = 7, *P* = 0.016).

**P*–values are based on likelihood ratio test

***P*–values are based on likelihood ratio tests between the multivariate regression models with and without the respective variable. The multivariate core model included a random intercept at the unit of the school and the categorical exposure variables sex, age group (8–11 years and 12–14 years), socioeconomic status, and project region, which were set a priori as potential confounders. All the other variables were assessed one by one and retained for the maximal model if their *P*–value was < 0.2. The final model was then obtained using backward selection with the same level of 0.2.
