# Supplementary material for: Prevalence of intestinal parasitic infections and associated risk factors among schoolchildren in the Plateau Central and Centre-Ouest regions of Burkina Faso
Source: Parasit Vectors. 2016 Oct 18;9:554. doi: 10.1186/s13071-016-1835-4 (PMC5069922; doi:10.1186/s13071-016-1835-4)
Supplement: Additional file 2: Table S2. — Results from univariate and multivariate logistic regression analysis for parasitic infection. (DOCX 76 kb) [file 13071_2016_1835_MOESM2_ESM.docx]

**Additional file 2: Table S2.** Results from univariate and multivariate logistic regression analysis for parasitic infection

| **Risk factor** | ***Hymenolepis nana***  **(*n* = 25)** | | | | | | | ***Schistosoma haematobium***  **(*n* = 15)** | | | | | | | **Intestinal pathogenic protozoa**  **(*n* = 290)** | | | | | | |
| --- | --- | --- | --- | --- | --- | --- | --- | --- | --- | --- | --- | --- | --- | --- | --- | --- | --- | --- | --- | --- | --- |
|  | Univariate log. regression* | | | | Multivariate log.  regression** | | | Univariate log. regression* | | | | Multivariate log.  regression** | | | Univariate log. regression* | | | | Multivariate log.  regression** | | |
|  | N^f^ | OR | 95% CI | *P* | aOR | 95% CI | P | N^f^ | OR | 95% CI | *P* | aOR | 95% CI | *P* | N^f^ | OR | 95% CI | *P* | aOR | 95% CI | *P* |
| **Sex** |  |  |  |  |  |  |  |  |  |  |  |  |  |  |  |  |  |  |  |  |  |
| Male (197) | 14 | 1.00 |  |  |  |  |  | 8 | 1.00 |  |  |  |  |  | 146 | 1.00 |  |  |  |  |  |
| Female (188) | 11 | 0.81 | 0.35–1.88 | 0.62 | * |  |  | 7 | 0.82 | 0.28–2.43 | 0.73 | * |  |  | 144 | 1.17 | 0.72–1.90 | 0.52 | * |  |  |
| **Age group** |  |  |  |  |  |  |  |  |  |  |  |  |  |  |  |  |  |  |  |  |  |
| 8–11 yrs (251) | 13 | 1.00 |  |  |  |  |  | 8 | 1.00 |  |  |  |  |  | 182 | 1.00 |  |  |  |  |  |
| 12–14 yrs (143) | 12 | 1.32 | 0.53–3.28 | 0.55 | * |  |  | 7 | 0.01 | 0.33–3.81 | 0.84 | * |  |  | 108 | 1.45 | 0.84–2.52 | **0.19** | 1.30 | 0.75–2.24 | 0.35 |
| **Region** |  |  |  |  |  |  |  |  |  |  |  |  |  |  |  |  |  |  |  |  |  |
| Centre-Ouest (187) | 20 | 1.00 |  |  |  |  |  | 7 | 1.00 |  |  |  |  |  | 160 | 1.00 |  |  |  |  |  |
| Plateau Central (198) | 5 | 0.20 | 0.05–0.80 | **0.02** | 0.32 | 0.09–1.15 | 0.08 | 8 | 2.65 | 0.04–160.76 | 0.64 | * |  |  | 130 | 0.32 | 0.18–0.57 | **< 0.001** | 0.33 | 0.18–0.58 | **< 0.001** |
| **Hygiene behaviour^a^** |  |  |  |  |  |  |  |  |  |  |  |  |  |  |  |  |  |  |  |  |  |
| Middle third (2) (227) | 14 | 1.00 |  |  |  |  |  | 12 | 1.00 |  |  |  |  |  | 174 | 1.00 |  |  |  |  |  |
| Lower third (1) (56) | 1 | 0.33 | 0.04–2.66 | 0.30 | * |  |  | 0 | na |  |  | * |  |  | 41 | 0.79 | 0.38–1.64 | 0.53 | * |  |  |
| Higher third (3) (102) | 10 | 1.63 | 0.68–3.92 | 0.28 | * |  |  | 3 | 0.45 | 0.12–1.72 | 0.24 | * |  |  | 75 | 0.84 | 0.48–1.47 | 0.54 | * |  |  |
| **Drinking water for consumption^b^** |  |  |  |  |  |  |  |  |  |  |  |  |  |  |  |  |  |  |  |  |  |
| From home (239) | 17 | 0.99 | 0.37–2.68 | 0.99 | * |  |  | 10 | 1.41 | 0.42–4.73 | 0.57 | * |  |  | 179 | 1.05 | 0.62–1.78 | 0.87 | * |  |  |
| From school (322) | 15 | 0.25 | 0.07–0.85 | **0.03** | 0.37 | 0.15–0.91 | **0.02** | 11 | 0.33 | 0.07–1.62 | **0.17** | 0.31 | 0.06–1.61 | 0.16 | 246 | 1.17 | 0.55–2.46 | 0.68 | ***** |  |  |
| **Water risk behaviours** |  |  |  |  |  |  |  |  |  |  |  |  |  |  |  |  |  |  |  |  |  |
| No water contact (93) | 7 | 1.00 |  |  |  |  |  | 1 | 1.00 |  |  |  |  |  | 70 | 1.00 |  |  |  |  |  |
| Playing (5) | 0 | na |  |  | * |  |  | 0 | na |  |  | * |  |  | 3 | 0.32 | 0.05–2.21 | 0.25 | * |  |  |
| Fishing (25) | 0 | na |  |  | * |  |  | 0 | na |  |  | * |  |  | 18 | 0.83 | 0.29–2.32 | 0.72 | ***** |  |  |
| Making laundry (56) | 3 | 0.60 | 0.14–2.58 | 0.49 | * |  |  | 1 | 1.68 | 0.10–29.40 | 0.72 | * |  |  | 40 | 0.81 | 0.37–1.78 | 0.60 | * |  |  |
| Domestic chores (206) | 15 | 0.69 | 0.24–1.94 | 0.48 | * |  |  | 13 | 7.38 | 0.89–61.15 | **0.06** | 2.04 | 1.05–3.97 | **0.04** | 159 | 1.07 | 0.58–1.97 | 0.84 | ***** |  |  |
| Any water contact^b^ (292) | 18 | 0.59 | 0.22–1.60 | 0.30 | * |  |  | 14 | 5.11 | 0.63–41.76 | **0.13** | 5.21 | 0.62–44.11 | 0.13 | 220 | 0.96 | 0.54–1.71 | 0.88 | ***** |  |  |
| **Sanitary practices children** |  |  |  |  |  |  |  |  |  |  |  |  |  |  |  |  |  |  |  |  |  |
| Using latrines at school (307) | 15 | 1.00 |  |  |  |  |  | 12 | 1.00 |  |  |  |  |  | 231 | 1.00 |  |  |  |  |  |
| Using latrines at home/teacher’s (7) | 1 | 3.73 | 0.35–39.37 | 0.27 | * |  |  | 0 | na |  |  | * |  |  | 5 | 1.31 | 0.23–7.60 | 0.61 | ***** |  |  |
| Open defaecation at  school^c^ (71) | 9 | 1.49 | 0.42–5.30 | 0.54 | * |  |  | 3 | 2.21 | 0.50–9.76 | 0.29 | * |  |  | 54 | 0.83 | 0.39–1.77 | 0.62 | ***** |  |  |
| **Caregiver’s education** |  |  |  |  |  |  |  |  |  |  |  |  |  |  |  |  |  |  |  |  |  |
| Never went to school (288) | 18 | 1.00 |  |  |  |  |  | 8 | 1.00 |  |  |  |  |  | 220 | 1.00 |  |  |  |  |  |
| Primary education (59) | 4 | 0.97 | 0.30–3.12 | 0.96 | * |  |  | 6 | 4.48 | 1.34–14.95 | **0.02** | 1.32 | 0.60–2.91 | 0.49 | 47 | 1.24 | 0.81–2.54 | 0.55 | ***** |  |  |
| Secondary education (38) | 3 | 1.58 | 0.41–6.17 | 0.51 | * |  |  | 1 | 0.72 | 0.08–6.49 | 0.77 | * |  |  | 23 | 0.62 | 0.29–1.32 | 0.22 | * |  |  |
| **Caregiver’s occupation^b^** |  |  |  |  |  |  |  |  |  |  |  |  |  |  |  |  |  |  |  |  |  |
| Agriculture (344) | 23 | 1.00 |  |  |  |  |  | 15 | 1.00 |  |  |  |  |  | 264 | 1.00 |  |  |  |  |  |
| Civil service (8) | 0 | na |  |  |  |  |  | 0 | na |  |  | * |  |  | 4 | 0.45 | 0.10–1.98 | 0.29 | * |  |  |
| Merchant (9) | 1 | 1.63 | 0.18–15.11 | 0.67 | * |  |  | 0 | na |  |  | * |  |  | 8 | 2.34 | 0.27–20.41 | 0.44 | * |  |  |
| Others^d^ (24) | 1 | 0.43 | 0.05–3.53 | 0.43 | * |  |  | 0 | na |  |  | * |  |  | 14 | 0.39 | 0.15–0.97 | **0.004** | 0.74 | 0.55–0.99 | **0.05** |
| **Animals^b^** |  |  |  |  |  |  |  |  |  |  |  |  |  |  |  |  |  |  |  |  |  |
| Possession of domestic animals (371) | 25 | na |  |  | * |  |  | 15 | na |  |  | * |  |  | 281 | 1.28 | 0.40–4.17 | 0.68 | * |  |  |
| Animals held in the house (246) | 19 | 1.76 | 0.67–4.62 | 0.25 | * |  |  | 9 | 0.75 | 0.24–2.29 | 0.61 | * |  |  | 186 | 1.00 | 0.61–1.64 | 0.99 | * |  |  |
| **Household sanitary conditions** |  |  |  |  |  |  |  |  |  |  |  |  |  |  |  |  |  |  |  |  |  |
| Traditional latrine (213) | 3 | 1.00 |  |  |  |  |  | 5 | 1.00 |  |  |  |  |  | 61 | 1.00 |  |  |  |  |  |
| No latrines/ open defaecation (83) | 19 | 1.84 | 0.47–7.25 | 0.38 | * |  |  | 9 | 2.61 | 0.29–23.73 | 0.39 | * |  |  | 164 | 1.08 | 0.58–2.00 | 0.81 | * |  |  |
| Improved latrine (89) | 3 | 0.96 | 0.18–5.16 | 0.97 | * |  |  | 1 | 3.03 | 0.33–28.13 | 0.33 | * |  |  | 65 | 1.21 | 0.59–2.48 | 0.60 | * |  |  |
| Soap for handwashing available^b^ (118) | 4 | 0.50 | 0.16–1.55 | 0.23 | * |  |  | 2 | 0.24 | 0.05–1.14 | **0.07** | 0.21 | 0.04–1.05 | 0.06 | 87 | 1.05 | 0.62–1.77 | 0.87 | * |  |  |
| **Household drinking water rainy season** |  |  |  |  |  |  |  |  |  |  |  |  |  |  |  |  |  |  |  |  |  |
| Tap source (37) | 3 | 1.00 |  |  |  |  |  | 3 | 1.00 |  |  |  |  |  | 23 | 1.00 |  |  |  |  |  |
| Borehole water (249) | 7 | 0.31 | 0.07–1.36 | **0.12** | 1.81 | 0.96–3.40 | 0.07 | 6 | 0.51 | 0.10–2.70 | 0.43 | * |  |  | 188 | 1.55 | 0.68–3.57 | 0.30 | * |  |  |
| Well (87) | 12 | 1.45 | 0.31–6.72 | 0.64 | * |  |  | 4 | 0.75 | 0.11–5.07 | 0.76 | * |  |  | 67 | 1.21 | 0.46–3.19 | 0.70 | * |  |  |
| Rain water, surface  water (12) | 3 | 2.85 | 0.38–21.33 | 0.31 | * |  |  | 2 | 8.62 | 0.56–132.44 | **0.12** | 1.75 | 0.68–4.51 | 0.25 | 12 | na |  |  | * |  |  |
| **Dry season** |  |  |  |  |  |  |  |  |  |  |  |  |  |  |  |  |  |  |  |  |  |
| Tap source (34) | 2 | 1.00 |  |  |  |  |  | 3 | 1.00 |  |  |  |  |  | 22 | 1.00 |  |  |  |  |  |
| Borehole water (261) | 9 | 0.53 | 0.10–2.79 | 0.45 | * |  |  | 7 | 0.57 | 0.11–2.98 | 0.50 | * |  |  | 198 | 1.21 | 0.49–2.98 | 0.68 | * |  |  |
| Well (81) | 12 | 2.10 | 0.33–13.30 | 0.43 | * |  |  | 4 | 0.76 | 0.11–5.20 | 0.78 | * |  |  | 61 | 0.77 | 0.26–2.31 | 0.68 | * |  |  |
| Surface water (9) | 2 | 3.33 | 0.01–0.30 | 0.33 | * |  |  | 1 | 1.95 | 0.11–36.16 | 0.65 | * |  |  | 9 | na |  |  | * |  |  |
| **Household drinking water storage** |  |  |  |  |  |  |  |  |  |  |  |  |  |  |  |  |  |  |  |  |  |
| Open^b^ (278) | 21 | 2.04 | 0.65–6.36 | 0.22 | * |  |  | 15 | na |  |  | * |  |  | 213 | 1.34 | 0.79–2.29 | 0.28 | * |  |  |
| Pot or canary (290) | 18 | 1.00 |  |  |  |  |  | 13 | 1.00 |  |  | * |  |  | 218 | 1.00 |  |  |  |  |  |
| Basin or bowl (16) | 2 | 1.31 | 0.25–6.87 | 0.75 | * |  |  | 0 | na |  |  | * |  |  | 14 | 1.75 | 0.37–8.23 | 0.48 | * |  |  |
| Canister (plastic jerrican) (59) | 4 | 1.49 | 0.44–5.00 | 0.52 | * |  |  | 0 | na |  |  | * |  |  | 47 | 1.27 | 0.62–2.60 | 0.52 | * |  |  |
| **Household drinking water treatment^b^** |  |  |  |  |  |  |  |  |  |  |  |  |  |  |  |  |  |  |  |  |  |
| Prior to consumption^e^ (69) | 7 | 1.18 | 0.44–3.20 | 0.74 | * |  |  | 4 | 1.13 | 0.32–4.05 | 0.85 | * |  |  | 51 | 0.79 | 0.41–1.50 | 0.46 | * |  |  |
| **Water contamination households^b^** |  |  |  |  |  |  |  |  |  |  |  |  |  |  |  |  |  |  |  |  |  |
| Coliform bacteria (89) | 2 | na |  |  | * |  |  | 3 | 0.38 | 0.02–6.62 | 0.51 | * |  |  | 69 | 3.30 | 0.56–19.56 | **0.19** | 0.96 | 0.12–7.63 | 0.97 |
| *Escherichia coli* (61) | 2 | na |  |  | * |  |  | 1 | 0.25 | 0.02–3.11 | 0.28 | * |  |  | 50 | 2.25 | 0.81–6.25 | **0.12** | 1.11 | 0.32–3.87 | 0.87 |
| Faecal streptococci (88) | 2 | na |  |  | * |  |  | 2 | 0.09 | 0.01–1.32 | **0.08** | 0.11 | 0.01–2.73 | 0.18 | 69 | 5.30 | 0.92–30.35 | **0.06** | 2.41 | 0.32–18.06 | 0.39 |
| Safe to drink (34) | 0 | na |  |  | * |  |  | 0 | na |  |  | * |  |  | 0 | na |  |  |  |  |  |
| **Water contamination children’s drinking cups^b^** |  |  |  |  |  |  |  |  |  |  |  |  |  |  |  |  |  |  |  |  |  |
| Coliform bacteria (101) | 6 | 0.18 | 0.01–4.61 | 0.30 | * |  |  | 4 | na |  |  | * |  |  | 78 | 1.13 | 0.28–4.52 | 0.86 | * |  |  |
| *Escherichia coli* (55) | 4 | 0.70 | 0.11–4.46 | 0.71 | * |  |  | 3 | 3.29 | 0.33–32.61 | 0.31 | * |  |  | 42 | 0.93 | 0.39–2.24 | 0.88 | * |  |  |
| Faecal streptococci (101) | 7 | na |  |  | * |  |  | 3 | 0.31 | 0.02–3.94 | 0.37 | * |  |  | 78 | 1.13 | 0.28–4.52 | 0.86 | * |  |  |
| Safe to drink (61) | 0 | na |  |  | * |  |  | 0 | na |  |  | * |  |  | 2 | 0.59 | 0.05–6.76 | 0.67 | * |  |  |
| **Water contamination community sources^b^** |  |  |  |  |  |  |  |  |  |  |  |  |  |  |  |  |  |  |  |  |  |
| Coliform bacteria (13) | 1 | 1.92 | 0.11–33.41 | 0.66 | * |  |  | 0 | na |  |  | * |  |  | 12 | 1.09 | 0.09–13.31 | 0.95 | * |  |  |
| *Escherichia coli* (9) | 1 | 3.38 | 0.19–60.24 | 0.41 | * |  |  | 0 | na |  |  | * |  |  | 9 | na |  |  | * |  |  |
| Faecal streptococci (10) | 1 | 2.89 | 0.16–51.13 | 0.47 | * |  |  | 0 | na |  |  | * |  |  | 10 | na |  |  | * |  |  |
| Safe to drink (15) | 1 | 0.67 | 0.04–11.56 | 0.78 | * |  |  | 0 | na |  |  | * |  |  | 21 | 0.71 | 0.06–8.66 | 0.79 | * |  |  |

^a^A new variable for hygiene behaviour was created using factor analysis with the mode and frequency of handwashing. Children were classified into three categories with poor, middle and good hygiene behaviours.

^b^The odds ratio (OR) refers to the comparison “yes” *vs* “no”

^c^Open defaecation includes the category of defaecating in the bush and behind the latrines

^d^‘Others’ includes homemakers, retirees and unemployed people

^e^Households reported to treat their drinking water through filtration and sedimentation

^f^N = positive cases

**P*–values are based on likelihood ratio tests

***P*–values are based on likelihood ratio tests between the multivariate regression models with and without the respective variable. The multivariate core model included a random intercept at the unit of the school and the categorical exposure variables sex, age group (8–11 years and 12–14 years), socioeconomic status, and project region, which were set a priori as potential confounders. All the other variables were assessed one by one and retained for the maximal model if their *P*–value was < 0.2. The final model was then obtained using backward selection with the same level of 0.2.
